# Elder abuse victimization patterns: latent class analysis using perpetrators and abusive behaviours

**DOI:** 10.1186/s12877-019-1111-5

**Published:** 2019-04-23

**Authors:** Ana João Santos, Baltazar Nunes, Irina Kislaya, Ana Paula Gil, Oscar Ribeiro

**Affiliations:** 1 Instituto de Ciências Biomédicas Abel Salazar, Universidade do Porto, Porto, Portugal; 2 Departamento de Epidemiologia, Instituto Nacional de Saúde Dr. Ricardo Jorge, Av. Padre Cruz, 1649-016 Lisboa, Portugal; 3 CISP - Centro de Investigação em Saúde Pública, Escola Nacional de Saúde Pública, Lisboa, Portugal; 4 CICS.NOVA - Centro Interdisciplinar de Ciências Sociais, Faculdade de Ciências Sociais e Humanas da Universidade Nova de Lisboa, Av. de Berna, 26-C, 1069-061 Lisboa, Portugal; 5 Departamento de Educação e Psicologia, Universidade de Aveiro, Aveiro, Portugal; 6 CINTESIS - Centro de Investigação em Tecnologias e Serviços de Saúde, Universidade do Porto, Rua Dr. Plácido da Costa, 4200-450 Porto, Portugal

**Keywords:** Elder abuse, Victimization, Perpetrators, Latent class analysis (LCA)

## Abstract

**Background:**

Research on elder abuse has defined it as a multidimensional construct that encompasses a set of different abusive behaviours, victims, perpetrators and settings. The array of possible elder abuse configurations is difficult to capture. This study sought to identify victimization patterns that represent distinct elder abuse configurations based on specific abusive behaviours and on the relationship with the perpetrator; it also sought to determine the association between these latent classes with victims’ characteristics.

**Method:**

Data comes from two elder abuse surveys: a representative sample of community-dwelling adults and a convenience sample of older adults reporting elder abuse to four state and NGOs institutions. Latent Class Analysis (LCA) was used to categorize victimization in the population-based (*N* = 245) and in the victims’ sample (*N* = 510) using 7 items measuring physical, psychological and financial abuse, and appointed perpetrators. Association tests were conducted to determine differences and similarities of victims’ characteristics between the different obtained classes.

**Results:**

The LCA procedure identified six different latent classes of victimization experiences in each of the samples, which were statistically and plausibly distinct. In the population-based survey: verbal abuse by others (29%); psychological abuse from children/grandchildren (18%); overlooked by others (18%); stolen by others (15%); verbal Intimate Partner Violence (IPV) (14%) and physical and psychological IPV (6%). In the victims’ survey: physical abuse by children/grandchildren (29%); physical IPV (26%); psychological abuse by children/grandchildren (18%); polyvictimization by others (16%); physical abuse by others (6%) and physical and psychological IPV (4%). In the victims survey the 6 groups significantly differ in age, gender, civil status, living arrangements, perceived social support and functional status.

**Conclusions:**

The results support the possibility of the multidimensionality of elder abuse not being accounted by the “classical” abuse typologies. Elder abuse victims seeking help may represent a distinct group from that included in population-based prevalence studies. The appointed perpetrators may be the most meaningful and relevant aspect in distinguishing victimization experiences. Further research is needed to develop tailored interventions to specific elder abuse cases and enhance successful outcomes.

**Electronic supplementary material:**

The online version of this article (10.1186/s12877-019-1111-5) contains supplementary material, which is available to authorized users.

## Background

Elder abuse has been gaining public, state and scientific attention for the past 40 years [1, 2]. Research has proliferated in the nineties with prevalence studies developed at regional and national levels [3, 4]. At the same time, even though not at the same rate, some conceptual and theoretical framework has been advanced [4]. Within a decade, two major reviews disclosed a wide variation between the phenomenon’s prevalence estimates, ranging from 1 to 36.2% [5, 6]. The difference between studies on conceptual and operational definitions, number and types of abuse included, study designs, and data collection methods could account for such variation [3, 5, 6].

Some of the differences on the conceptual and operational definitions of elder abuse can be attributed to the phenomenon’s complexity, which includes different victims, perpetrators and contexts [1, 7]. Presently two different definitions are more common according to the geographic region. In Europe, the World Health Organization’s adopted definition [8] has been widely used, which states that “Elder abuse is a single or repeated act or lack of appropriate action, occurring within any relationship where there is an expectation of trust which causes harm or distress to an older person” (p. 152). In the USA The Panel to Review Risk and Prevalence of Elder Abuse and Neglect [9] developed another definition that includes: “(a) intentional actions that cause harm or create a serious risk of harm, whether or not intended, to a vulnerable elder by a caregiver or other person who stands in a trust relationship to the elder; (b) failure by a caregiver to satisfy the elder’s basic needs or to protect the elder from harm” (p. 39).

These definitions are comprehensive enough to include the usually considered four types of elder abuse (psychological, physical, financial, sexual) and neglect, different settings (community and institutional) and different perpetrators (e.g., family members, close friend, colleagues, paid workers). However, such broadness also represents difficulties in defining, characterizing and explaining a phenomenon that has many possible configurations.

The multidimensionality of elder abuse recognises abusive behaviours, victims, perpetrators and contexts as elements that define victimization experiences, all of which are necessary to characterize and explain its occurrence. This is to say that addressing elder abuse is not just about the older person. Research, theory and practice also need to consider the abusive behaviours and the history of the relationship with the perpetrator. For instance, financial abuse perpetrated by a formal caregiver or committed by an adult-child may configure distinct victimisations experiences, with different motivations, risk factors and consequences. Some evidence does suggest pronounced differences of risk factors, case characteristics and interpersonal dynamics across single and co-occurring types of abuse, but also across perpetrators within the same type of abuse [10, 11].

However, most research still characterises elder abuse focusing on a single dimension (e.g., type of abuse) [9, 12]. This “monolithic” perspective, which tries to assess a multidimensional problem as a unit, has hampered the development of appropriated theories [9, 12]. In fact, recent research indicates elder abuse to be a set of disparate events that are not always related [12], and Bonnie and Wallace [9] had even proposed that in some cases it can correspond to independent occurrences with different determinants and explanations.

The characterisation and description of elder abuse as “monolithic” and the tendency of over inclusion all its possible configurations affects the value of research evidence, measures, social policies, clinical tools and/or interventions guidelines. Existing typologies do not properly address the different victimization experiences in terms of risk factors, determinants and perpetrators [13–15]. Aware of this issue, studies have developed approaches according to specific types of elder abuse rather than focusing on the overall construct [14, 16–19].

Despite these recent approaches, studies considering, simultaneously, more than one of elder abuse dimensions (e.g., victim, perpetrator and context) are not notable. Elder abuse comprises the interplay of distinct dimensions and blanket approaches are therefore unlikely to address its complex nature.

This study assumes that elder abuse is better described in its multidimensional nature and aims to provide a more complex representation of the victimisation experiences by incorporating more than one of the problems’ dimensions in a description of possible configurations. The question was whether there was any way of capturing a more complex representation of elder abuse, encompassing more than one dimension and if this representation could better represent the phenomenon.

The present study focuses on configurations of victimization experiences considering both abusive behaviours and appointed perpetrators. Drawing on data from two elder abuse surveys, it uses Latent Class Analysis (LCA) to categorize abuse occurrence into subgroups. Based on the individuals’ shared characteristics or behaviours, LCA uses observed data to group individuals into latent classes, in this case the positive or negative answer to specific abusive behaviours and indication (or not) of a specific perpetrator. We selected this approach because we believe that there might be diverse victimization experiences, even within the same abuse type, and because empirical data on the phenomenon presentation might help underpin the variation across these different types of elder abuse. We hypothesize that victimization experiences will classify differently from the traditional abuse type typology and that these will be distinctively associated with victims’ characteristics.

## Methods

### Study design and setting

This paper involves a secondary analysis of data from two cross-sectional surveys conducted as part of the Ageing and Violence study [20], which targeted community-dwelling elder abuse. One of the surveys was a population-based study aimed at estimating elder abuse prevalence (i.e., physical, psychological, financial and sexual and neglect) within a representative sample of Portuguese community-dwelling individuals aged 60 and over. The second survey intended to characterize adults aged 60 or more years reporting the abuse to governmental and non-governmental institutions [21]. This last survey encompassed a convenience sample obtained from individuals reporting to one of four institutions: a non-governmental organization aimed at support of domestic violence victims; a welfare state organization; the public security police; and the national forensic and legal medicine institute. The summary of the sampling, setting and data collection procedure of these two surveys is presented in Table 1; a more complete description has been reported elsewhere [20, 21].

**Table 1:** Population-based and victims’ surveys description (sample and data collection)

		Population- based survey	Victims’ survey
Sample	Inclusion criteria	Being 60 or more years of age, Having land or mobile telephone, Living in private households, Living in Portugal for the past 12 months	Being 60 or more years of age, Living in private households, Living in Portugal for the past 12 months
	Sampling	Nationally representative probability sample stratified by seven geographic regions with homogeneous allocation of sampling units	Convenience sample of individuals using the services of four institutions
	Sample size	1123	510
Data collection	Method	Telephone interviews	Face-to-face interviews
	Instruments	Structured questionnaire	Structured questionnaire
	Time period	October 2012	November 2011 to March 2013

The present analysis focused on older adults self-reporting experiences of psychological, physical or financial abusive behaviours in the two surveys. From the population-based survey, 245 older adults answered positively to at least one of the questions assessing these three forms of abuse. In the victims’ survey, all individuals (*N* = 510) reported having experienced at least one physical, psychological or financial abuse behaviour.

### Measures

Both surveys employed a standardized questionnaire that included sociodemographic information, health and functional status, social and economic variables in addition to questions assessing physical, sexual, psychological, financial abuse and neglect.

Self-reported demographic variables included age (recoded into three age groups: 60–69 years, 70–79 years and 80 and more years), gender, civil status (single or divorced, married or currently cohabiting, widowed), living arrangements (alone, living with others) and years of schooling (up to 4 years of schooling, five or more years).

Health variables included reporting of at least one chronic disease and functional status. We used a typology of Activities of Daily Living (ADL) [22], divided in personal and instrumental activities, and assessed the need of assistance. In this specific study, only two categories were considered: not reliant on help with ADL, and reliant on help with ADL for at least one activity.

Perceived social support was assessed by asking participants “do you have enough people who you may ask for help and support when you face problems?”. From the five-possible close-ended responses (I have a lot people I can rely on; I have enough people I can rely on; I do not know if people will help when I’m in need; I have a few people I can rely on and I have no one I can rely on), we recoded the variable into two response categories: 1) plenty or enough people to rely on, and 2) few and no one available to rely on.

In both surveys, abuse and neglect were assessed by means of the same 12 behaviours [20]. For the present study only items with absolute counts higher than 10 were considered [20, 21] - a requirement for the implementation of the LCA. Hence, of the 12 abusive behaviours, only seven were included: 1) stealing or using property without consent; 2) undue household appropriation and not contributing to household expenses; 3) physical aggression; 4) hindering of speaking or meeting someone; 5) threatening; 6) verbal aggression, insulting, humiliating and 7) ignoring or refusal to talk.

For each of the positively answered abusive behaviours, respondents were asked to indicate a perpetrator, which included spouse/partners, children, grandchildren, sons and daughters-in-law, other family members, friends, neighbours, work colleagues, paid professionals and volunteers. If a participant reported more than one abusive behaviour, then he or she could indicate more than one perpetrator. In this study, the perpetrators variable was recoded into three main categories: spouse/partner; children/grandchildren and others. Respondents were also asked about the frequency of occurrence of each of these behaviours during the past 12 months.

### Statistical analysis

In each of the considered samples, we applied Latent Class Analysis to investigate whether seven behaviourally specific abusive items and the three categories of perpetrators cluster together and enable a plausible characterization of co-occurrence patterns capturing the phenomenon variation. An exploratory approach of the latent class measurement model was conducted on 245 participants from the population-based survey and on 510 participants from the victims’ survey.

We used the LCA Stata® Plugin (version 11 or higher) [23] to identify the number of distinct abuse subtypes (classes, k), the relative size of each subtype (proportion of self-reported victims within each class), and the distribution of characteristics within each subtype (probability of each of the items based on class membership, ρ) [24]. For the LCA analysis we used 10 binary items: 7 abusive behaviours and 3 perpetrators categories.

The number of classes was determined by the entropy measure, the log likelihood, the parsimony indices and the bootstrapped likelihood ratio test (BRLT) [25, 26]. The best model has higher entropy, lower values of Akaike information criterion (AIC), Bayesian information criterion (BIC) and SSABIC, a larger log likelihood and a G^2^ value that is significantly smaller than the G^2^ value of the k − 1 model based on the BLRT results. In addition, because victimization experience groups should be plausible, the conceptual suitability and precision of the classes was qualitatively assessed. Following these procedures, the computed membership probability allowed assigning each participant to one of defined classes. Optimal fit considered average posterior probabilities above 70% [27].

Using the chi-square and exact Fisher tests for significance, the final latent groups were compared in terms of age groups, civil status, living arrangements, schooling, perceived social support, chronic disease and functional status. The significance level for all analysis was set at 5%.

## Results

### Samples description

Table 2 lists the characteristics of both samples. The distribution of these characteristics was rather similar for both surveys. Most of participants were women (75.1% for the population-based; 77.2% for the victims’ surveys). Approximately half was in the youngest age group (53.1% for the population-based and 50.3% for the victims’ surveys in the 60 to 69 years old age group) and was married or living in civil union (58.2 and 61.9%, respectively). Living with others was the most common situation for participants in both surveys (73.2% for the population-based and 89.6% for the victims’ surveys), as was having up to 4 years of schooling (80.2 and 85.9%, respectively). The majority reported at least one chronic health condition (82.0% for the population-based and 76.3% for the victims’ surveys), but no need of assistance in ADL (80.4 and 77.0%, respectively).

**Table 2:** Sociodemographic and health characteristics of participants in two surveys of elderly persons

	Population-based survey N = 245	Victims’ survey N = 510
	n(%)	n(%)
Sex		
Women	184 (75.1)	382 (77.2)
Men	61 (24.9)	113 (22.8)
Age groups		
[60–69 years]	130 (53.1)	249 (50.3)
[70–79 years]	78 (31.8)	175 (35.4)
[80 + years]	37 (15.1)	71 (14.3)
Civil status		
Single	13 (5.3)	14 (2.9)
Married/Civil Union	132 (58.2)	301 (61.9)
Divorced/Separated	20 (8.2)	58 (11.9)
Widow	69 (28.3)	113 (23.3)
Living arrangements		
Alone	65 (26.8)	53 (10.4)
With others	178 (73.2)	457 (89.6)
Schooling		
Up to 4 years of schooling	190 (80.2)	429 (85.9)
5 years or more	47 (19.8)	70 (14.1)
Social support		
Plenty/ enough	137 (58.8)	281 (55.7)
Few/ No one	96 (41.2)	223 (44.3)
Chronic disease		
Yes	201 (82.0)	374 (76.3)
No	44 (18.0)	116 (23.7)
ADL		
Yes	48 (19.6)	117 (23.0)
No	197 (80.4)	392 (77.0)

The frequencies of abusive behaviours and perpetrators observed in each of the surveys are presented in Table 3, which correspond to the 10 items selected for the LCA Model. Two behaviours encompassed in psychological abuse were the most frequently reported by participants in the population-based survey: verbal aggression (46.5%) and ignoring or refusing to talk (43.6%). In the victims’ survey, the most often reported behaviour was physical aggression (84.7%), followed by verbal aggression (62.0%). In the population-based survey the most common appointed perpetrators were individuals outside the nuclear family (62.5%), whereas the nuclear family was reported as being responsible for most abusive experiences in the victims’ survey (48.2% for spouse or partners and 42.3% for children/grandchildren).

**Table 3:** Abusive behaviours and perpetrators distribution reported by the participants from the two surveys of elderly persons

		Population-based survey	Victims’ survey
		n(%)	n(%)
Physical	Physical aggression	36 (14.8)	430 (84.7)
	Hindering of speaking or meeting someone	19 (7.8)	113 (22.5)
Psychological	Threaten to abandon, harm, punish or institutionalize	48 (19.7)	194 (38.6)
	Verbal aggression, insulting, humiliating	113 (46.5)	315 (62.0)
	Ignoring or refusal to talk	105 (43.6)	182 (36.6)
Financial	Stealing or using property without consent	65 (26.5)	180 (36.4)
	Undue household appropriation	19 (7.8)	129 (25.8)
Perpetrator^a^	Spouse/ partner	60 (26.8)	243 (48.2)
	Children/grandchildren	47 (21.0)	213 (42.3)
	Other	140 (62.5)	48 (9.5)
	N	245	510

^a^Multiple perpetrators could be reported

### LCA application to population-based survey

The six-class solution was the chosen model for classifying victimization experiences in the population-based sample based on the following statistical criteria (fit statistics for models 3 through 6 are presented Additional file 1). Classification certainty (entropy) was marginally greater for the three and the six-class solution (92%). AIC, G^2^ values and SSABIC values decreased from three to six-class solution. Also, the BLRT results showed a statistically significant improvement in model fit (*p* < .05) from the three to the six-class solution and it was not significant when comparing the six to the seven-class solution. The six-class model not only had the best fit and highest level of separation, as it was also interpretable and presented distinctive patterns. The results of the six-class solution are presented in Table 4. Victimization experiences were assigned descriptive labels based on the predominant abusive behaviours and main perpetrators.

**Table 4:** Items response probabilities (population-based survey)

	Verbal by others		Psychological by children/ grandchildren		Overlooked by others		Stolen by others		Verbal IPV		Physical and psychological IPV	
	29%(*N* = 70)		18%(*N* = 44)		18%(N = 44)		15%(*N* = 38)		14%(*N* = 35)		6%(*N* = 14)	
	P	SE	P	SE	P	SE	P	SE	P	SE	P	SE
Physical aggression	0.18	0.05	0.09	0.04	< 0.005	< 0.005	< 0.005	< 0.005	0.23	0.07	0.78	0.14
Hindering of speaking/meeting	0.03	0.02	0.05	0.03	0.03	0.03	0.06	0.04	0.13	0.06	0.50	0.14
Threaten	0.29	0.06	0.29	0.07	0.01	0.02	< 0.005	0.01	0.01	0.05	0.98	0.05
Verbal aggression	0.77	0.06	0.82	0.08	0.01	0.02	0.01	0.03	0.71	0.08	0.73	0.14
Ignoring or refusing to speak	0.38	0.06	0.73	0.08	0.99	0.03	0.08	0.11	0.15	0.07	0.30	0.13
Stealing	0.12	0.04	0.35	0.07	0.02	0.09	0.99	0.02	0.03	0.03	0.21	0.11
Undue household appropriation	0.16	0.05	0.07	0.04	0.06	0.04	< 0.005	0.00	0.06	0.04	< 0.005	0.01
Spouse/partner	0.17	0.05	< 0.005	0.01	< 0.005	0.01	0.02	0.01	0.99	0.01	0.99	0.03
Children/ grandchildren	< 0.005	0.01	0.99	0.01	< 0.005	< 0.005	< 0.005	0.01	< 0.005	0.01	0.22	0.11
Other	0.99	< 0.005	0.18	0.06	0.99	0.01	0.99	0.01	0.02	0.06	0.01	0.04

Verbal abuse by others was the largest class (29.0%), with a high probability of individuals reporting to have experienced verbal aggression (0.77) from someone other than their nuclear family (0.99). Psychological abuse from children/grandchildren (Class 2) comprised 18.0% of the sample. In this class, abuse is characterized by verbal aggression (0.82) and ignoring or refusing to talk (0.71) perpetrated by children/grandchildren and almost no probability of physical aggression (0.09). Being overlooked (Class 3) and being stolen (Class 4) comprise only one abusive behaviour each and were perpetrated outside the nuclear family. Being overlooked by others included individuals with a high probability of being ignored or refused to be talked to (0.99) and very low probability of any other abusive behaviour (< 0.07), whereas Class 4 included individuals with very high probability of being stolen (0.99) and, again, very low probability of any other abusive behaviour (< 0.09). Intimate Partner Violence (IPV) in older age was found for two classes: verbal IPV (15.0%) and physical and psychological IPV (6.0%). The last comprised individuals with a high probability of reporting both physical and verbal aggression (0.78 and 0.73, respectively) and being threatened (0.98) by their partner/spouse.

Table 5 presents the distribution of participants’ sociodemographic and health characteristics by the different obtained groups. To understand if there were differences in the distribution of participants in each of the groups by sex, age group, civil status, schooling, living arrangements, perceived social support, functional and health status, the chi-square and exact Fisher tests were used.

**Table 5:** Sociodemographic and health variables of victims, conditional on class membership (N = 245)

		Verbal by others (n = 70)	Psychological by children (n = 44)	Overlooked by others (N = 44)	Stolen by others (N = 38)	Verbal IPV (N = 35)	Physical and psychological IPV (N = 14)	*p*-value
Sex	Female	77.1	70.5	70.5	63.2	85.7	100.0	^a^
	Male	22.9	29.5	29.5	36.8	14.3	0.00	
Age group	60–69	52.9	52.3	50.0	42.1	65.7	64.3	< 0.05
	70–79	41.4	22.7	34.1	29.0	25.7	28.6	
	80+	5.7	25.0	15.9	29.0	8.6	7.1	
Civil Status	Single	11.4	0.0	4.6	7.9	0.0	0.0	^a^
	Married/ Civil Union	57.1	54.6	52.3	52.6	79.4	57.1	
	Divorced/ Separated	10.0	6.8	6.8	2.6	14.7	7.1	
	Widow	21.4	38.6	36.4	36.8	5.9	35.7	
Schooling	Up to 4 years	80.9	76. 2	83.3	75.0	77.1	100.0	^a^
	5 or more years	19.1	23.8	16.7	25.0	22.9	0.0	
Living arrangements	Alone	33.3	20.9	27.3	36.8	11.4	21.4	0.115
	With others	66.7	79.1	72.7	63.2	88.6	78.6	
Perceived social support	Plenty/ Enough	64.2	43.9	61.9	63.9	60.6	50.0	0.355
	Few/No one	35.8	56.1	38.1	36.1	39.4	50.0	
Chronic disease	Yes	81.4	86.4	77.3	86.8	77.1	85.7	0.789
	No	18.6	13.6	22.7	13.2	22.9	14.3	
AVD	Yes	24.3	20.5	9.1	29.0	14.3	14.3	0.213
	No	75.7	79.6	90.9	71.0	85.7	85.7	

Note: ^a^ Due to cells frequencies count with zero, the association test wasn’t performed

The only significant difference was in age group distribution. Psychological abuse from children/grandchildren and stealing by others presented a higher proportion of individuals from the oldest age group (25.0 and 29.0%, respectively). Due to small sample size and cells frequencies count with zero, the association test wasn’t performed for sex, civil status and schooling. However, we observe a higher proportion of women in all classes, and a lower proportion of men specifically in the two classes characterising IPV in old age. Both these groups also present a higher proportion of married/living in civil union individuals and no single individuals. No other variable was found to be significantly associated with class membership. In all classes, most individuals lived with others, reported at least one chronic disease, and were independent in ADLs. Half or little more than half reported having plenty or enough people to rely on.

### LCA application to the victim’s survey

The six-class solution was also the chosen model for classifying victimization experiences in the victims’ sample based on the following statistical criteria (fit statistics for models 3 through 7 are presented Additional file 1).

Classification certainty (entropy) was marginally greater for the three and four-class solutions (92%). AIC and G^2^ values decreased from three to six- or seven-class solution. There was an important drop in the BIC and SSABIC values from three to five-class solution; however, the SSABIC remained similar in the five, six and seven-class solution. The BLRT results showed a statistically significant improvement in model fit (*p* < .05) from three to seven-class solution. Comparing the six to seven-class solution, BLRT was still significant but to a lesser degree (*p* = .04). Further inspection showed that for the seven-class solution, one of the classes presented a posterior probability of 0.1. Additionally, the conditional probabilities of the items in the six-class solution were more plausible than the seven-class solution. Given the negligible difference between six and seven-class models in terms of the SSABIC and G^2^ Values, and following the parsimonious principle, the six-class model was chosen. The results of the six-class solution for the victims’ sample are presented in Table 6. Victimization experiences classes were assigned descriptive labels based on the predominant abusive behaviours and main perpetrators.

**Table 6:** Items response probabilities (victims’ survey), conditional on class membership (*N* = 510)

	Physical by children/ grandchildren		Physical IPV		Physical and psychological by children		Polyvictimization by others		Physical abuse by others		Physical and psychological IPV	
	29%(*N* = 148)		26%(*N* = 133)		18%(*N* = 96)		16%(*N* = 85)		6%(*N* = 29)		4%(*N* = 19)	
	P	SE	P	SE	P	SE	P	SE	P	SE	P	SE
Physical aggression	0.87	0.04	0.87	0.04	0.79	0.04	0.74	0.12	0.85	0.08	0.79	0.03
Hindering of speaking/meeting	0.10	0.03	0.22	0.05	0.19	0.04	0.33	0.12	0.05	0.05	0.35	0.04
Threaten	0.01	0.03	0.08	0.03	0.61	0.05	0.57	0.16	0.01	0.03	0.66	0.06
Verbal aggression	0.03	0.06	0.25	0.10	0.96	0.04	0.84	0.13	0.08	0.10	0.99	0.01
Ignoring or refusing to speak	0.16	0.04	0.16	0.04	0.50	0.05	0.48	0.15	0.02	0.05	0.56	0.05
Stealing	0.31	0.06	0.20	0.05	0.55	0.05	0.63	0.13	0.31	0.10	0.32	0.04
Undue household appropriation	0.24	0.05	0.15	0.04	0.42	0.04	0.23	0.11	0.10	0.06	0.23	0.04
Spouse/partner	< 0.005	< 0.005	0.99	< 0.005	< 0.005	< 0.005	0.04	0.02	0.02	0.01	0.99	< 0.005
Children/grandchildren	0.99	< 0.005	< 0.005	< 0.005	0.99	< 0.005	0.03	0.01	0.02	0.01	< 0.005	< 0.005
Other	< 0.005	< 0.005	< 0.005	< 0.005	< 0.005	< 0.005	0.99	0.02	0.99	0.01	< 0.005	< 0.005

Physical abuse by children/grandchildren was the largest class. It comprised 29.0% of the sample and individuals with high probability of reporting physical aggression (0.87) perpetrated by children/grandchildren (0.99). Physical Intimate Partner Violence (IPV) (Class 2) comprised 26.0% of the sample. In this class, abuse is characterized by physical aggression (0.87) perpetrated by a spouse or a partner. Physical and psychological abuse by children/grandchildren was the third largest class comprising 18% of the sample. Within this class, individuals tend to report physical aggression (0.79), threatening behaviours (0.91) and verbal aggression (0.66) perpetrated by children/grandchildren. For some items, this class has lower homogeneity when compared with others: there is similar probability of individuals reporting to have or not experienced being ignored (0.50), stolen (0.50) or having someone appropriated the household or not contributed to household expenses (0.42). The “polyvictimization by others” class, which comprised 16.0% of the sample, included physical aggression (0.74), verbal aggression (0.84), and stealing (0.63). It also presented low homogeneity for the other two psychological abusive behaviours. There is similar probability of individuals in this class to have or have not experienced threatening behaviours (0.57) or being ignored (0.48). Class 5 was labelled “physical abuse by others” because victims in this class have a high probability of reporting physical aggression and very low probability of reporting any other abusive behaviour (< 0.32). The last class - physical and psychological IPV – comprise 4.0% of the sample and includes individuals reporting to have experienced physical aggression (0.74), threatens (0.66) and verbal aggression (0.99) by the spouse/partner.

Table 7 presents the distribution of participants’ sociodemographic and health characteristics by the different obtained groups. To understand if there were differences in the distribution of participants in each of the groups by sex, age group, civil status, schooling, living arrangements, perceived social support, functional and health status, the chi-square and exact Fisher tests were used.

**Table 7:** Sociodemographic and health variables of victims, conditional on class membership (N = 510)

		Physical by children	Physical IPV	Physical and psychological by children	Polyvictimization by others	Physical abuse by others	Physical and psychological IPV	*p*-value
Sex	Female	88.5	70.7	74.0	69.4	62.1	79.0	< 0.001
	Male	11.5	29.3	26.0	30.6	37.9	21.0	
Age group	60–69	71.0	29.3	64.6	36.5	41.4	26.3	< 0.001
	70–79	23.0	46.6	32.3	40.0	34.5	47.4	
	80+	6.0	24.1	3.1	23.5	24.1	26.3	
Civil Status	Single	3.1	0.7	1.1	0.0	10.3	31.6	^a^
	Married/ Civil Union	39.2	88.9	74.7	47.6	44.8	26.3	
	Divorced/Separated	9.2	9.0	17.9	13.1	10.3	10.5	
	Widow	48.5	1.4	6.3	39.3	34.5	31.6	
Schooling	Up to 4 years	84.5	91.5	76.3	89.0	86.2	94.4	0.038
	5 or more years	15.5	8.5	23.7	11.0	13.8	5.6	
Living arrangements	Alone	6.8	6.8	17.7	9.4	20.7	15.8	0.017
	With others	93.2	93.2	82.3	90.6	79.3	84.2	
Perceived social support	Plenty/ Enough	57.8	46.9	69.5	47.6	58.6	63.2	0.012
	Few/No one	42.2	53.1	30.53	52.4	41.4	36.8	
Chronic disease	Yes	82.1	72.4	77.2	73.2	72.0	73.7	0.432
	No	17.9	27.6	22.8	26.8	28.0	26.3	
AVD	Yes	16.9	25.6	8.4	47.4	48.3	31.8	< 0.001
	No	83.1	74.4	91.6	52.6	51.7	68.2	

Note: ^a^ Due to cells frequencies count with zero, the association test wasn’t performed

Regarding sex, all groups presented a higher proportion of female victims: the highest for physical abuse by children/grandchildren and the lowest for physical abuse by others.

There were more victims aged between 60 and 69 in the groups “physical abuse by children/grandchildren” and “physical and psychological by children/grandchildren”. These groups also comprised the lowest proportion of individuals aged 80 or more years. The oldest age group (80+ years) was more present in both classes of IPV (physical or physical and psychological IPV).

A higher proportion of married individuals was found in the “physical IPV” (88.9%) and the “physical and psychological by children/grandchildren” (74.7%) groups, whereas the highest proportion of widows was observed in the “physical abuse by children/grandchildren” (48.5%) and “polyvictimization by others” (39.3%) groups. The highest proportion of single individuals was found in the “physical and psychological IPV” group (31.6%).

A larger proportion of individuals in the “polyvictimization by others” group lived alone, in comparison with victims in the groups “physical abuse by children/grandchildren” (6.8%) and the “physical IPV” (6.8%). The number of participants having plenty or enough people to rely on was higher in the victims from the “physical and psychological by children/grandchildren” (69.5%) and the “physical and psychological IPV” (63.2%) groups. More individuals from the “physical abuse by others” (48.3%) and “polyvictimization by others” (47.4%) groups reported limitations in ADLs. People from the “physical and psychological by children/grandchildren” group were mainly independent in their ADLs (91.6%).

## Discussion

This study sought to describe patterns of victimization experiences using two surveys of older persons: a representative sample of community-dwelling adults and a convenience sample of older adults reporting elder abuse to four state and NGOs institutions. The LCA procedure identified six different latent classes of victimization experiences in each of the samples, which were statistically and plausibly distinct, based on the presence or absence of abusive behaviours and appointed perpetrators.

The results support the likelihood of elder abuse being a multidimensional phenomenon that is not accounted by the “classical” abuse typologies and highlight three distinct aspects. First, elder abuse victims seeking help and individuals included in population-based studies of elder abuse prevalence may represent quite distinct groups. Second, the appointed perpetrators and some abusive behaviours may be more meaningful and relevant aspect in distinguishing victimization experiences. Finally, the victims’ survey indicates distinct elder abuse case profiles.

On the first aspect, the two LCA model results underline different victimization configurations in the population-based and in the victims’ surveys. The victims’ survey comprised experiences of abuse perpetrated mostly by elements of the nuclear family (e.g., spouse/partner and children/grandchildren). Psychological abuse was more common in the population-based survey, whereas physical abuse was more frequent in the victims’ survey. Also, polyvictimization was only found in this victims’ survey LCA model, comprising physical aggression (0.74), verbal aggression (0.84), and stealing (0.63) perpetrated by individuals outside the nuclear family. Both polyvictimization and physical abuse point out to more severe victimization experiences in the victims’ survey. Differences between population-base studies and studies employing services/clinical samples have been acknowledged in elder abuse research [5, 6]. Burnes and colleagues, for instance, observed that older adults experiencing more types and severe forms of abuse perceived their situation as more serious, suggesting that these victims were more likely to seek support [10]. Victims might undervalue or “perceive” psychological abuse as not being “serious enough” to ask for help [28, 29]. At the same time, services might be more prompt to respond and intervene in cases of physical abuse. In fact, despite being less frequent, physical abuse is often given greater clinical relevance by experts when compared to emotional abuse or neglect [30]. Overall, the two LCA models presented in our study may represent two distinct groups of abuse victims that display different victimizations experiences, and this requires distinct responses. Isolated abusive behaviours seem to be more common when perpetrated by others, while co-occurring abusive behaviours characterise violence that takes place within the family.

The second noteworthy finding was that data from the application of the LCA to elder abuse configurations showed differences by perpetrator, but also by abusive behaviours. Differences were found regarding the relevance of abusive behaviours to the classes’ distinction, pointing out that some abusive behaviours committed by specific perpetrators might better distinguish elder abuse configurations. The perpetrators categories were one of the most distinctive items of the classes (with values close to 1 or close to 0). According to our findings, in either sample, each category of perpetrator was included in more than one class (exception was children/grandchildren in the population-based survey). The occurrence of distinct psychological, physical or financial abusive behaviours by the same category of perpetrators adds specificity to elder abuse characterization. The relationship dynamics between victim and perpetrator may be more accurate if specific abusive behaviours are taken into account [31]. On this matter, a clear distinction is made between abusive behaviours perpetrated by family members and individuals outside the nuclear family. In both surveys the groups that comprised abusive behaviours perpetrated by individuals outside the nuclear family were the ones that were statistically more distinct from others (“overlooked by others” and “stolen by others” in the prevalence survey and “polyvictimisation by others” in the victims survey). In the victims survey the groups of violence perpetrated by partners or spouses were also very distinctive from the other groups. In both surveys, the abusive behaviours that resulted in the least distinctive items were “hindering of speaking or meeting someone” and “undue household appropriation”. Financial abusive behaviours were also found to be not very distinctive, except for the group “stolen by others” (stealing) in the population-based survey. These results indicate that the family violence approach can be adequately employed to describe and understand some configurations of elder abuse. The abusive relationship is seen as the product of gender and age inequalities, where the perpetrator uses a pattern of coercive tactics to gain and maintain his/her power and control [32]. The unbalance of power might be particularly felt when dependency and care needs increase over time and the older adult must depend on the offspring in their day-to-day living [33, 34]. While this approach can be helpful to understand elder abuse perpetrated by spouse, partners or offspring, it doesn’t seem to be comprehensive enough to contextualize elder abuse perpetrated by individuals outside the nuclear family. It is important to consider what changes in old age may be precipitating factors associated with an increase vulnerability to elder abuse in what groups of older people. The results support the growing assumption that elder abuse is multifaceted, hardly fitting into one single unifying theory [7, 35, 36]. Rather than a single theory, the conceptual framework of elder abuse should consider the differentiate impact that specific factors can have according to the individuals and contexts involved. The ecological approach provides a useful framework to the multi-dimensions perspective and variability of elder abuse, because it promotes the inclusion of variables at the individual (victim and perpetrator) and the relationship levels, and variables related to broad ideological values and norms of a culture [37]. The ecological approach refuses the linear “adding up” version of other conceptual approaches to elder abuse as it proposes that the degree or impact of each risk and protective factor is mediated by individual, community and social level characteristics.

The same highlight is provided by the application of the LCA model to the victims’ sample and to the distinct elder abuse case profiles found. In the victims’ survey, the results suggest different elder abuse case profiles of older adults victimized by children or grandchildren. In the most prevalent group (29%) we mostly found older women living alone (88.3% of women and 48.5% of widowers) being physically abused by their children and grandchildren.

Other two classes indicates the expressive proportion of elder abuse between partners in later life, an already observed finding in elder abuse research [38]. IPV as part of elder abuse can be both conjugal violence grown old, where abuse that has begun earlier in life continues into older age and a new experience of abuse [7, 38, 39]. Despite presenting similar traits, victims in the groups “physical IPV” and “physical and psychological IPV”, may be representing “new” and “old” IPV. The “physical and psychological IPV” group may represent, in part, new relationships that have led to IPV – in this group we found a highest proportion of both single (31.6%) and divorced or separated (10.5%) individuals and widowers (31.6%).

Finally, the remaining two groups indicate profiles of elder abuse outside the nuclear family: polyvictimization by others and physical abuse by others. Compared to the other groups describing victimization within the nuclear family, these two groups share more similarities than differences. This suggests that despite the perpetrators category encompassing a very wide range of relationships, abuse by spouse or partner and by offspring is similar than abuse by someone outside the nuclear family. In addition and despite domestic violence and to some extent elder abuse has been defined as a more common feminine experience [38, 40, 41], these groups show that older males with dependency for their ADLs might be vulnerable to victimization experiences, particularly by individuals outside the nuclear family.

The study is not without limitations. The first regards the nature of the data of both samples: the cross-sectional designs are always subject to response bias and do not provide data on temporal relationships. Also, the victims’ survey was a convenience sample, which may also include a sample bias. Secondly, the data collection methods may not be the more adequate to capture victimization experiences from the most vulnerable group of older adults, such as those with physical or cognitive impairments. Thirdly, the low frequencies in some items obtained in the population-based sample did not allow exploring the association of the victims’ characteristics and class membership. Finally, victimization experiences groups identified in this study are item and sample dependent. Classifying victimization experiences using other data or including different indicators in the model may generate additional groups defined by distinct characteristics.

## Conclusion

Although elder abuse researchers have noted an association between abuse type and specific perpetrators [42], relatively few have sought to define victimization configurations according to the abusive behaviours and perpetrators. Data from the application of the Latent Class Analysis to elder abuse configurations showed differences between the two samples, with more severe forms of violence characterizing elder abuse within the family and isolated abusive behaviours more commonly perpetrated by individuals outside the family. The results also showed that some abusive behaviours and victims-perpetrators dyads might be more common and comprise “typical” elder abuse experiences. While interpersonal abuse (such as physical and verbal aggression) is a common abuse behaviour within victimisations experienced by spouses/partners or offspring, stealing is probably better accounted by perpetrators outside the nuclear family. Overall, there seems to be differences considering the type of elder abuse that occurs within and outside the nuclear family. Despite not being so severe (with concomitant occurrence of different abusive behaviours), violence perpetrated outside the family might be more hidden and different strategies than those employed to respond to family violence may be required. Given the distinctions and similarities between the groups, different approaches must be developed and implemented to tackle the elder abuse within and outside the family – blanket approaches to the problem will most likely not answer to the diversity of situations of violence against older adults.

## Additional file

Additional file 1: Presentation of fit statistics for models 3 through 6 (DOCX 23 kb)

## Abbreviations

ADL: Activities of Daily Living
AIC: Akaike information criterion
BIC: Bayesian information criterion
BRLT: Bootstrapped likelihood ratio test
IPV: Intimate Partner Violence
LCA: Latent Class Analysis
