# Supplementary material for: Elder abuse victimization patterns: latent class analysis using perpetrators and abusive behaviours
Source: BMC Geriatr. 2019 Apr 23;19:117. doi: 10.1186/s12877-019-1111-5 (PMC6480599; doi:10.1186/s12877-019-1111-5)
Supplement: Supplementary file 1 — Presentation of fit statistics for models 3 through 6 (DOCX 23 kb) [file 12877_2019_1111_MOESM1_ESM.docx]

**Latent Class Analysis models fit statistics**

The number of classes was determined by the entropy measure, the log likelihood, the parsimony indices and the bootstrapped likelihood ratio test (BRLT) [1, 2]. In addition, because abuse subtypes should be plausible, the conceptual suitability and precision of the classes was qualitatively assessed.

Entropy measures how well individuals are assigned to latent classes (class differentiation). It ranges from zero to one, with values closer to 1 indicating better differentiation. The log likelihood is a function of the observed responses in the sample conditional on the model parameters. The parsimony indices are measures of the goodness of fit of the model that consider number of parameters, the sample size and other factors. These include the Akaike Information Criterion (AIC) and the Bayesian Information Criterion (BIC) or adjusted BIC (SSABIC). The BLRT assesses the relative improvement in fit between a model with k classes versus a smaller model with k − 1 classes. The optimal class solution has high entropy, low AIC, BIC and adjusted BIC values, a larger loglikelihood and a G^2^ value that is significantly smaller than the G^2^ value of the k − 1 model based on the BLRT results. These criteria allowed for the best model to be selected.

**LCA application to population-based survey**

The six-class solution was the chosen model for classifying abusive experiences in the population-based sample. Classification certainty (entropy) was marginally greater for the three and the six-class solution (92%). AIC and G2 values decreased from three to six-class solution, except for four-class solution, where they increased. BIC values declined from four- through six-class solutions, while slightly lower in three-class solution. Because, BIC penalizes models with more parameters and thus provides an upper bound indicator for class selection [1], the Adjusted BIC (SSABIC) is, in many cases, recommended instead. In this case, the SSABIC was lower for the six-class solution model (Table 1). Finally, the BLRT results showed a statistically significant improvement in model fit (p <.05) from the three to the six-class solution and it was not significant when comparing the six to the seven-class solution. The six-class model not only had the best fit and highest level of separation, but it was also interpretable and presented distinctive patterns.

Table 1. LCA model for the population-based survey

| Number of classes | 3 | 4 | 5 | 6 |
| --- | --- | --- | --- | --- |
| Class membership probability | 55%, 25%, 20% | 18%, 27%, 28%, 27% | 15%, 18%, 19%, 28%, 21% | 15%, 14%, 18%, 29%, 6%, 18% |
| Log-likelihood | -1070.64 | -1092.14 | -1012.31 | -995.23 |
| G^2^ Values | 392.70 | 435.71 | 276.07 | 241.90 |
| Entropy | 92% | 85% | 91% | 92% |
| AIC | 456.71 | 521.72 | 384.07 | 371.90 |
| BIC | 568.75 | 672.27 | 573.14 | 599.49 |
| SSABIC | 467.31 | 535.96 | 401.96 | 393.43 |
| df | 991 | 980 | 969 | 958 |
| BRLT (*p*) | 3*4 (.01) | 4*5 (.01) | 5*6 (.01) | 6*7 (.08) |

**LCA application to victim’s survey**

The six-class solution was also the chosen model for classifying abusive experiences in the victims’ sample. Classification certainty (entropy) was marginally greater for the three and four-class solutions (92%) compared to five, six (91%) and seven-class solutions (90%). AIC and G2 values decreased from three to six or seven-class solution. There was an important drop in the BIC and adjusted BIC (SSABIC) values from three to five-class solution; however, the adjusted BIC remained similar in the five, six and seven-class solution. The BLRT results showed a statistically significant improvement in model fit (p <.05) from three to seven-class solution. Comparing the six to seven-class solution, BLRT was still significant but to a lesser degree (p = .04) (Table 2). Further inspection showed that for the seven-class solution, one of the classes presented a posterior probability of 0.1. The seventh abuse subtype that emerged comprised being threatened and verbal aggression (0.78 and 0.98, respectively), by the spouse or partner (0.72). The other two classes, from the six-solution solution, where the perpetrator was the spouse or partner presented a diminished frequency (from 24% to 15% and from 4% to 3%). Other aspect taken into consideration was the high homogeneity of characteristics/behaviours within each subtype. The conditional probabilities of the items in the six-class solution were more plausible than the seven-class solution. Finally, given the negligible difference between six and seven-class models in terms of the adjusted BIC and G2 Values, and following the parsimonious principle, the six-class model was chosen.

Table 2. LCA model for the victims’ survey

| Number of classes | 3 | 4 | 5 | 6 | 7 |
| --- | --- | --- | --- | --- | --- |
| Class membership probability | 24%, 48%, 28% | 10%, 20%, 29%, 42% | 10%, 20%, 28%, 17%, 26% | 6%, 26%, 28%, 17%, 26%, 4% | 6%, 18%, 4%, 17%, 26%, 3%, 26% |
| Log-likelihood | -2571.42 | -2480.86 | -2417.12 | -2399.65 | -2383.25 |
| G^2^ Values | 641.79 | 460.66 | 333.19 | 298.24 | 265.45 |
| Entropy | 92% | 92% | 91% | 91% | 90% |
| AIC | 705.79 | 546.66 | 441.19 | 428.24 | 474.45 |
| BIC | 841.29 | 728.74 | 669.85 | 703.48 | 739.26 |
| SSABIC | 739.71 | 592.25 | 498.45 | 497.15 | 498.03 |
| df | 991 | 980 | 969 | 958 | 947 |
| BRLT (*p*) | 3*4 (.01) | 4*5 (.01) | 5*6 (.01) | 6*7 (.04) | 7*8 (.07) |

References

1. Lanza S, Rhoades B. Latent class analysis: An alternative perspective on subgroup analysis in prevention and treatment. Prev Sci. 2013;14:157–68.

2. Lanza ST, Collins LM, Lemmon DR, Schafer JL. PROC LCA: A SAS Procedure for Latent Class Analysis. Struct Equ Model A Multidiscip J. 2007;14:671–94.
